# Immediate effect of abdominal muscle contraction on pelvic floor morphology in postpartum women

**DOI:** 10.3389/fmed.2026.1800064

**Published:** 2026-04-17

**Authors:** Yu Wang, Jiajia Ye, Yao Lu, Yan Zhuo, Jianqi Fang

**Affiliations:** 1 Department of Ultrasound, Fujian Maternity and Child Health Hospital, Fuzhou, Fujian, China; 2 Fujian Key Laboratory of Women and Children’s Critical Diseases Research [Fujian Maternity and Child Health Hospital (Fujian Women and Children’s Hospital)], Fuzhou, Fujian, China; 3 Department of Rehabilitation Assessment, Rehabilitation Hospital Affiliated to Fujian University of Traditional Chinese Medicine, Fuzhou, Fujian, China; 4 Department of Women’s Health Care, Fujian Maternity and Child Health Hospital, Fuzhou, Fujian, China; 5 Women’s Hospital, School of Medicine, Zhejiang University, Hangzhou, Zhejiang, China

**Keywords:** abdominal muscle, exercise, pelvic floor, pelvic floor muscle, ultrasound

## Abstract

**Objective:**

This study aimed to explore the immediate effect of abdominal muscle contraction on the pelvic floor morphology in postpartum women.

**Methods:**

This observational cross-sectional repeated-measures study recruited 90 participants who visited the Fujian Maternity and Child Health Hospital from May 2024 to March 2026. All participants underwent pelvic floor assessments using four-dimensional (4D) ultrasound in seven different positions.

**Results:**

The results showed a general decreasing trend in the hiatal area and retrovesical angle and an increasing trend in the urethral tilt angle during pelvic floor muscle contraction. The bladder neck position, cervical position, and rectal ampulla position descended during drawing-in (*Padj =* 0.005, *Padj* = 0.106, and *Padj* = 0.018, respectively), head-lift1 (*Padj =* 0.002, *Padj =* 0.032, and *Padj <* 0.001, respectively), head-lift2 (*Padj <* 0.001, *Padj <* 0.001, and *Padj <* 0.001, respectively), and 90 °flexion of the hips and knees (*Padj <* 0.001, *Padj =* 0.055, and *Padj =* 0.495, respectively). The differences remained significant for pelvic floor muscle contractions during the drawing-in, head-lift1, head-lift2, and one-leg lift for bladder neck positions (*Padj <* 0.001, *Padj =* 0.014, *Padj =* 0.023, and *Padj =* 0.033, respectively) and head-lift2 for the rectal ampulla position (*Padj <* 0.001). In contrast, the cervical position were higher than rest during pelvic floor muscle contractions in drawing-in (*Padj <* 0.001), head-lift1 (*Padj =* 0.011), one-leg lift (*Padj <* 0.001), and 90° flexion of the hips and knees (*Padj =* 0.006).

**Conclusion:**

Drawing-in, head-lift, and flexion of the hips and knees were associated with immediate pelvic organ descent and hiatal area enlargement in this study, with the head-lift2 maneuver showing a relatively higher mechanical loading degree. The simultaneous contraction of the pelvic floor muscles during these maneuvers correlated with an immediate reduction in pelvic organ descent.

## Introduction

Pelvic floor dysfunction (PFD) encompasses a spectrum of disorders including pelvic organ prolapse (POP), urinary dysfunction, and defecatory dysfunction (1). Hormonal fluctuations, mechanical stress during pregnancy, and pelvic floor trauma from vaginal delivery synergistically increase the susceptibility to PFD (2). Studies show that between 33 and 56% of reproductive-aged women worldwide experience PFD (3–5). While PFD is not inherently life-threatening, it exerts a profound negative impact on quality of life and exhibits strong associations with anxiety, depression, and surgical intervention in severe cases (6–8). Pelvic floor muscle (PFM) training remains the cornerstone of conservative intervention, aiming to enhance muscular strength and restore organ support (9).

Postpartum women frequently experience comorbidities with PFD, such as diastasis recti abdominis (DRA) and low back pain (10–13). Notably, no studies have systematically investigated the impact of abdominal muscle training protocols (abdominal breathing and head-lift exercises) on these multimodal postpartum conditions. While existing evidence supports the efficacy of abdominal muscle training for isolated DRA and low back pain (14, 15), its safety and therapeutic value in the context of concurrent PFD remain unestablished. This knowledge gap gives rise to a critical clinical problem: can abdominal muscle training be safely recommended for postpartum women with coexisting PFD? This study hypothesizes that pressure transmission between the pelvic and abdominal cavities is continuous, although the transmissions are anatomically compartmentalized by fascial structures. A study by Djivoh et al. demonstrated that the contraction of the rectus abdominis and transversus abdominis muscles increases intra-abdominal pressure (16). However, it remains unknown whether the elevated pressure leads to significant displacement and deformation of pelvic floor structures.

Therefore, the aims of this study are as follows: first, to evaluate the immediate effect of abdominal muscle contraction on the pelvic floor morphology; and second, to explore whether PFM contraction can protect pelvic floor structures from displacement and deformation caused by abdominal muscle contraction, using pelvic floor 4-dimensional (4D) ultrasound in postpartum women.

## Materials and methods

### Participants

This observational cross-sectional repeated-measures study included 90 participants who visited the Fujian Maternity and Child Health Hospital from May 2024 to Mar 2026. This study was approved by the Ethics Committee of the hospital (No. 2022YJ108) in accordance with the guidelines of the Declaration of Helsinki on 20 July 2023 and registered in the Chinese Clinical Trial Registry (ChiCTR2300073756). All patients underwent a standardized interview to assess their medical history (age, height, weight, BMI, neonatal weight, gestational weight gain, education, gestation, and parity) and an ultrasonic assessment of the pelvic floor. The inclusion criteria included participants who had a vaginal delivery for the index birth within 6 months and who could tolerate a gynecological examination. The exclusion criteria included: participants who had active vaginal bleeding during assessment, those who had a pelvic floor surgery history that may affect the results of the assessment, those who could not perform PFM contraction (no change in the hiatal area in ultrasonic imaging), and those who could not perform PFM contraction independently of abdominal muscles (which was detected by pelvic floor surface electromyography before ultrasonic testing) (17).

### Assessment of pelvic floor 4D ultrasound

**Figure 1:**
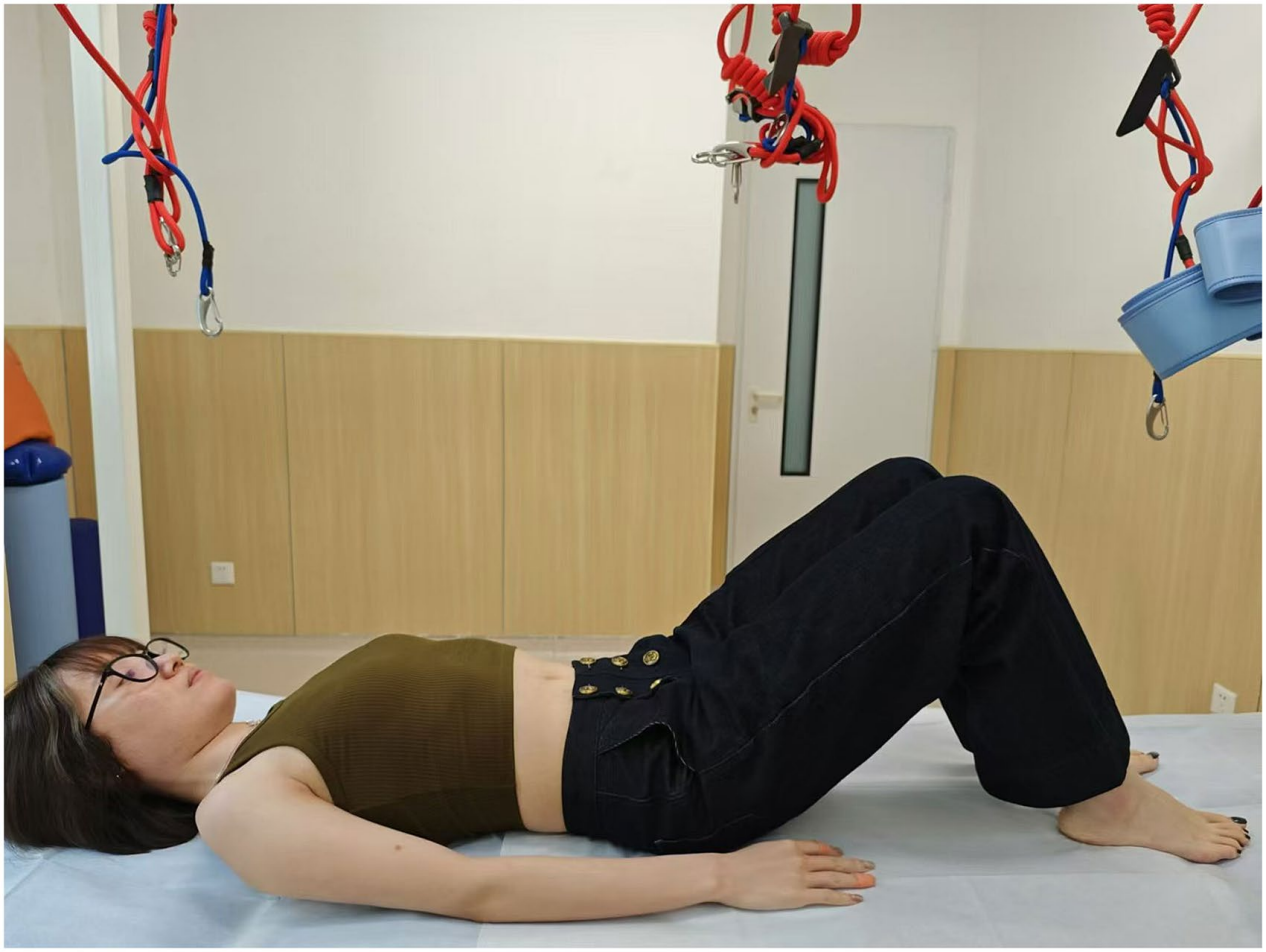
Lithotomy position (all these figures were demonstrated by Y.L.).

**Figure 2:**
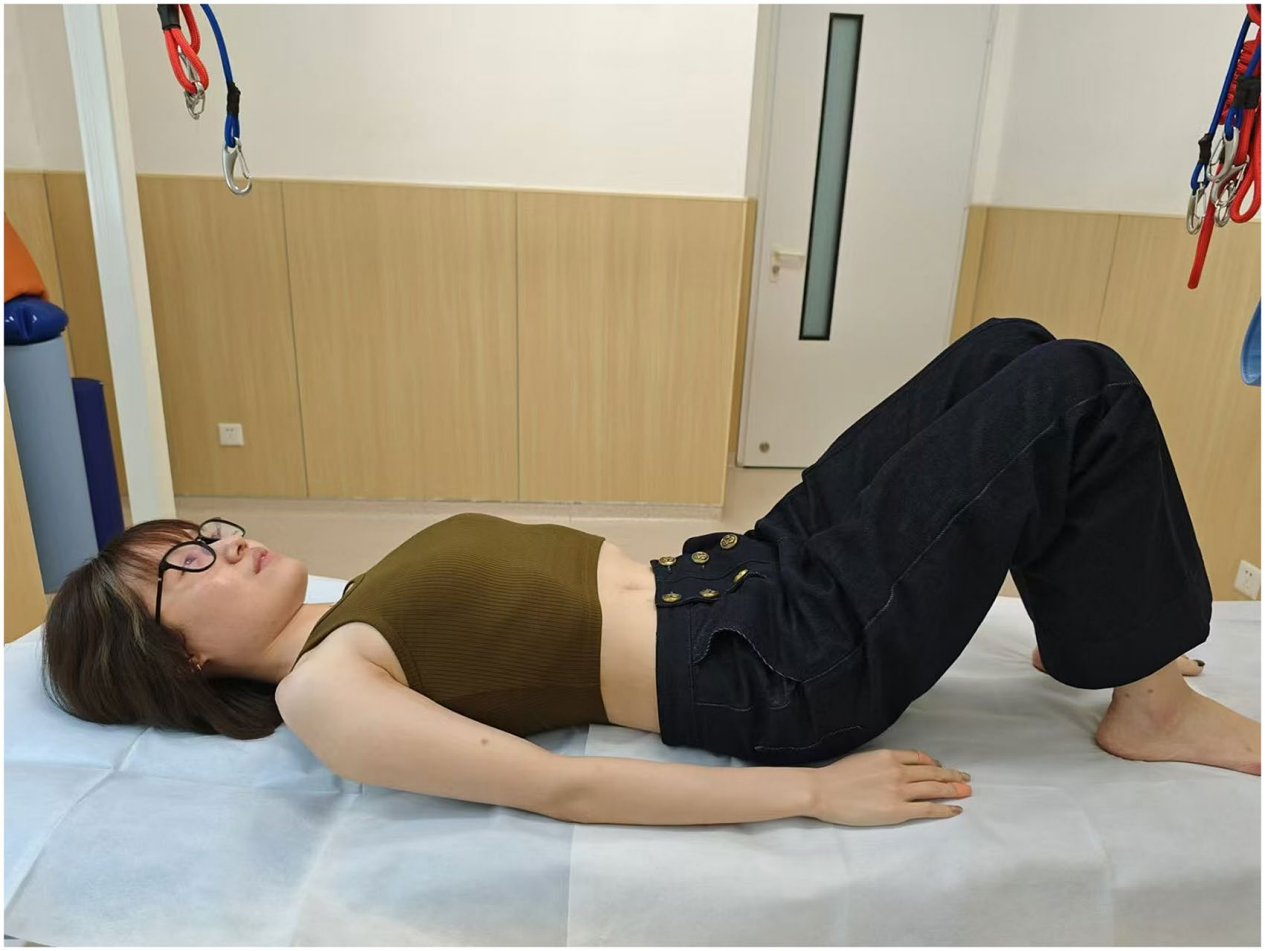
Drawing-in position.

**Figure 3:**
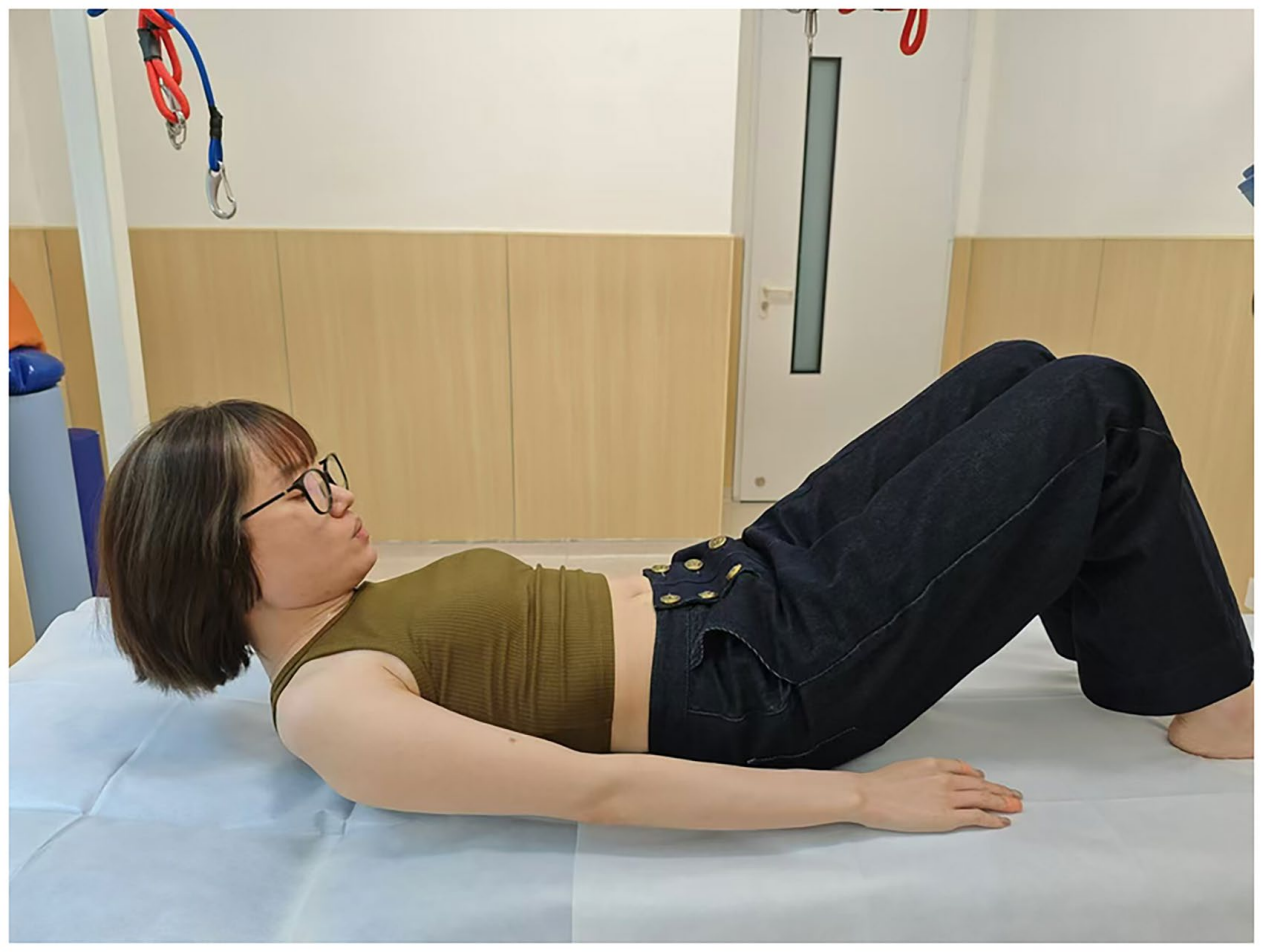
Head-lift1: head-lift (when the shoulder blades are about to leave the bed).

**Figure 4:**
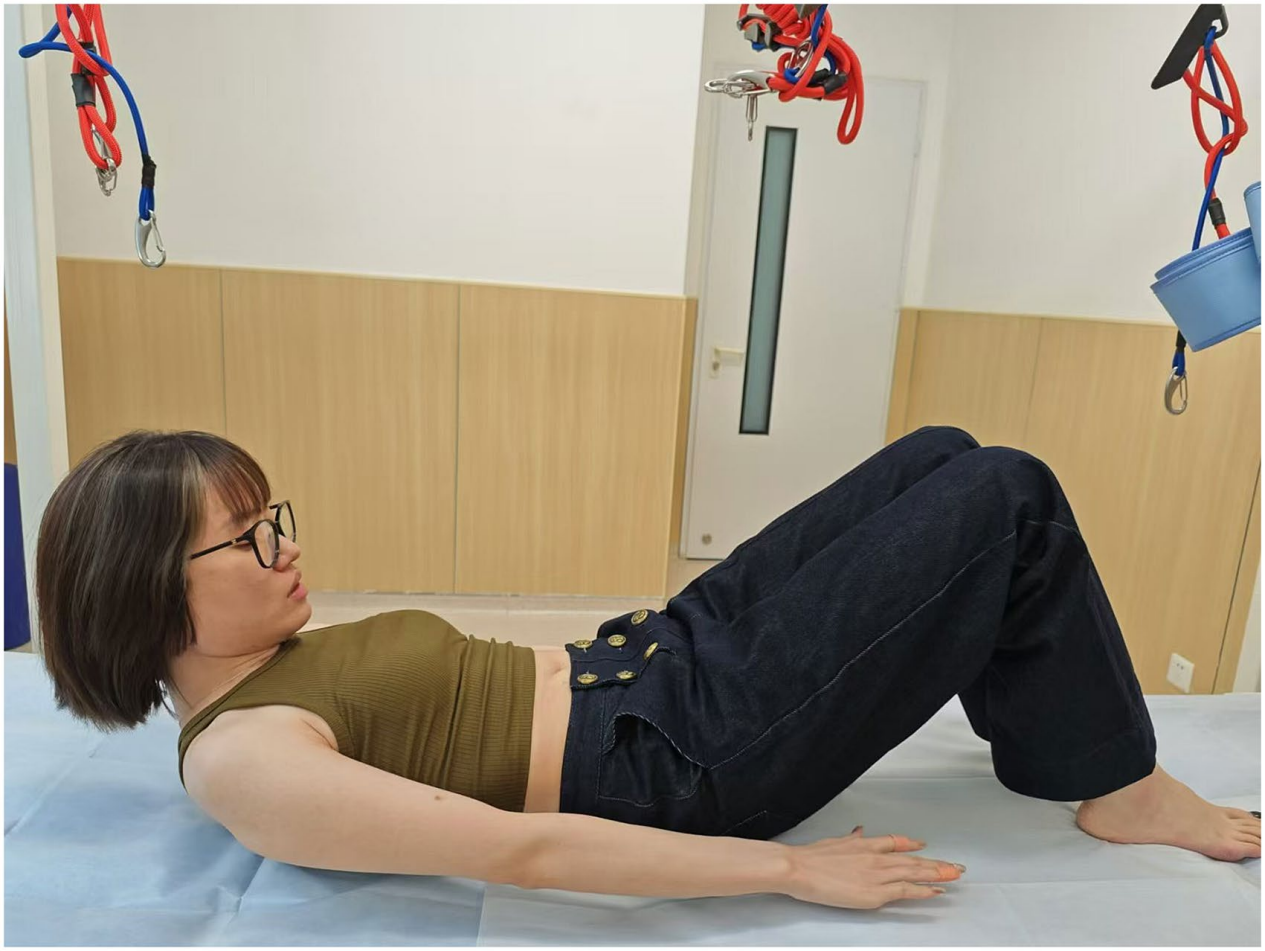
Head-lift2: head-lift (when the shoulder blades completely leave off the bed).

**Figure 5:**
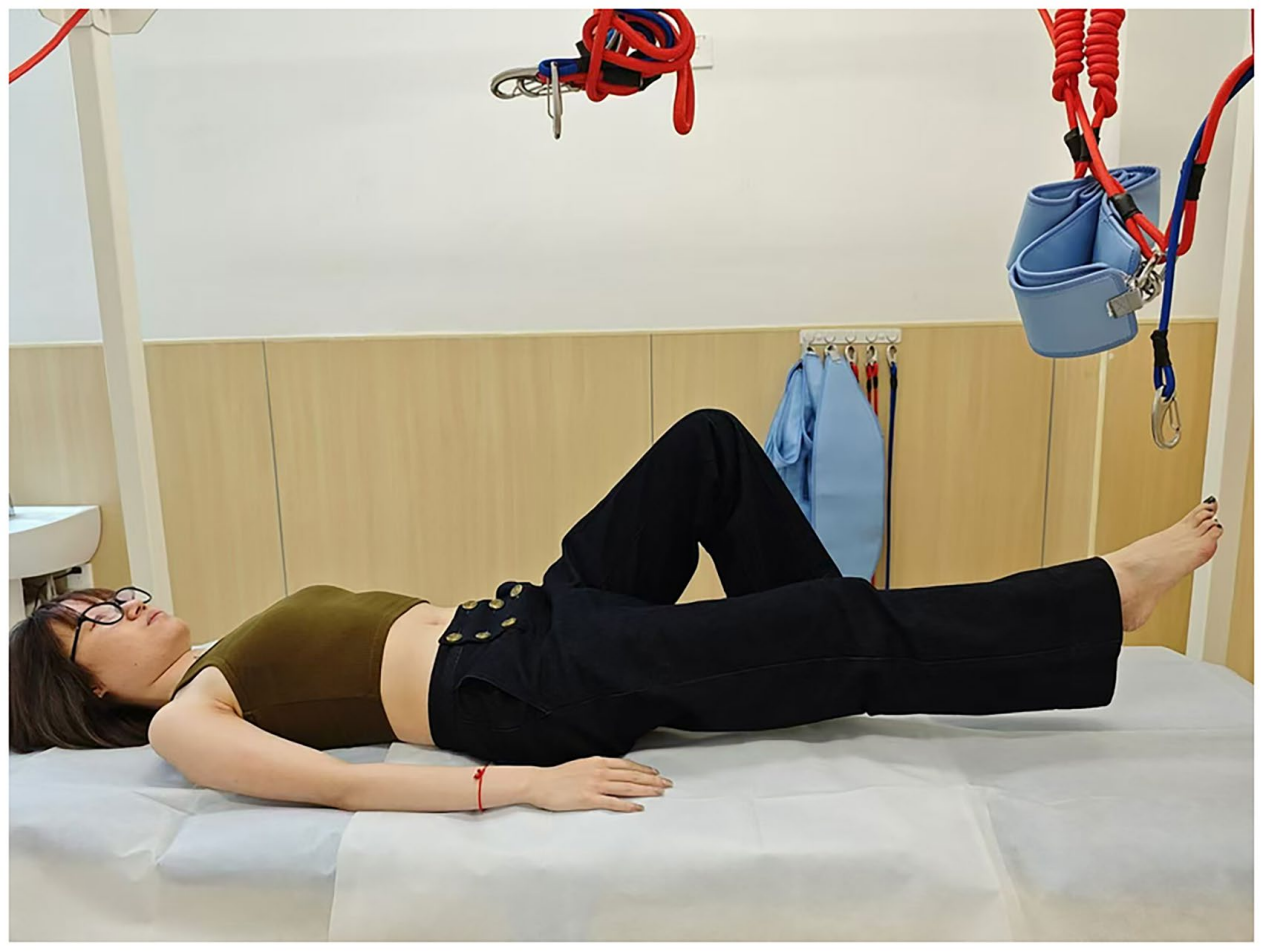
One leg lift: lithotomy position with one leg straightly hanging over the bed (randomly chose one leg).

**Figure 6:**
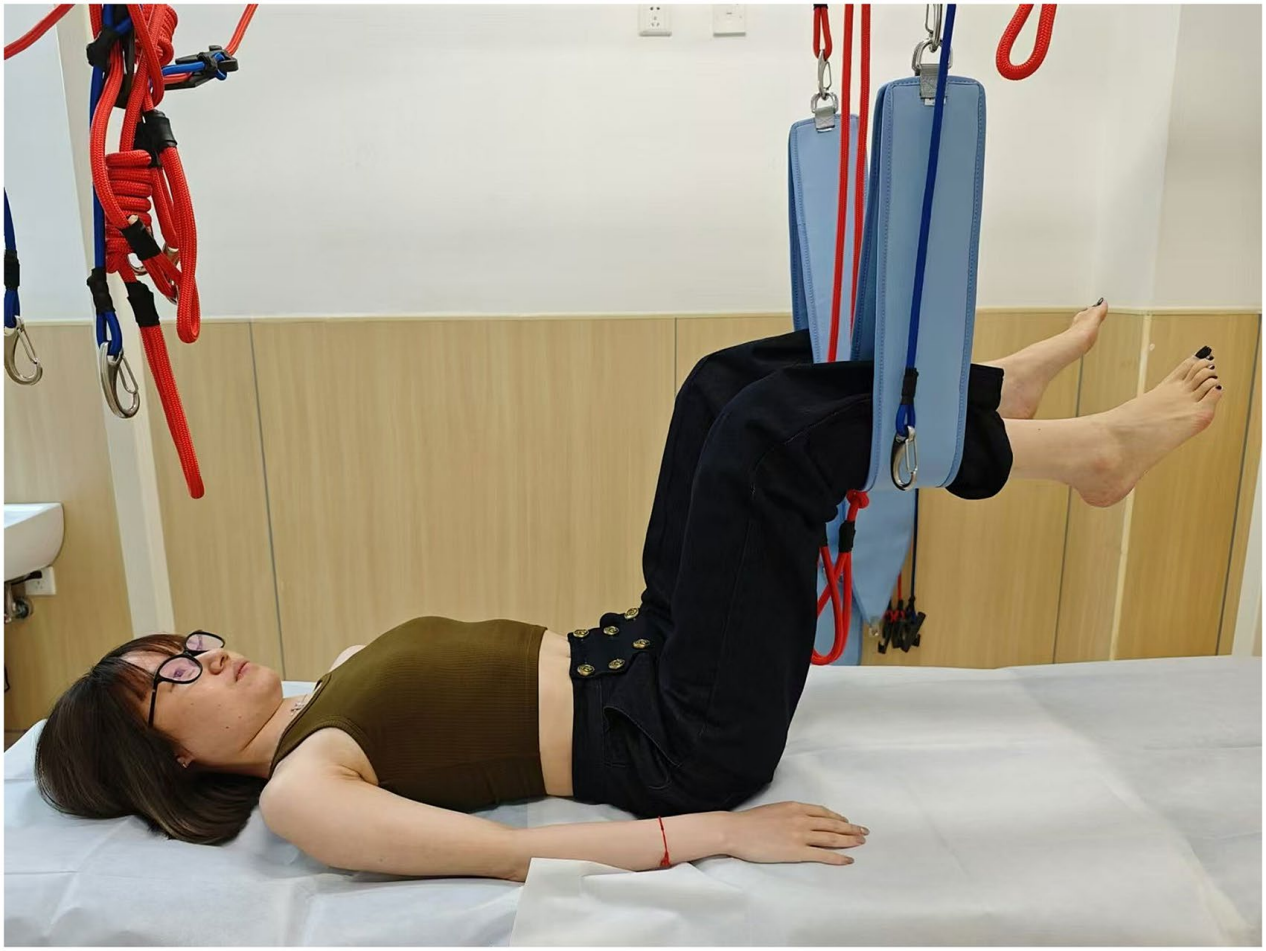
HKF(R): 90° hips and knees flexion and legs parallel to each other (there was a tape supporting the legs to keep the participants in a relaxed state).

**Figure 7:**
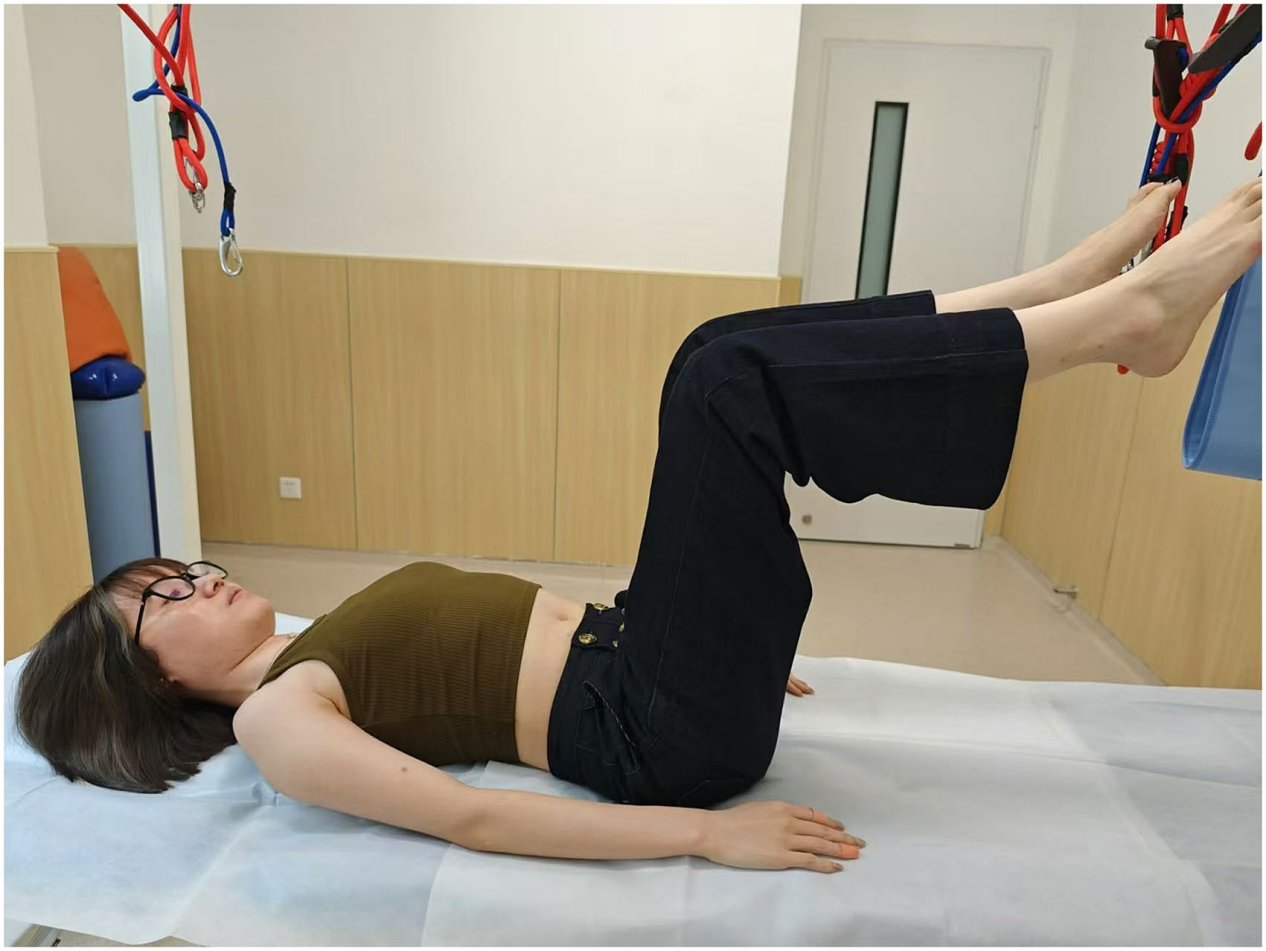
HKF(C): 90° hips and knees flexion and legs parallel to each other (no supporting and participants were asked to keep their waist as close to the bed as possible).

Pelvic floor four-dimensional (4D) ultrasound has been widely recommended for assessing pelvic floor morphology due to its non-invasiveness and objectivity, and it has also been proved to have a strong intra-group reliability (18–20). All ultrasound assessments were performed by one of the authors (Y. W.) who has more than 15 years of experience in ultrasound imaging. Assessment images were stored on a computer after being labeled with numerical codes (rather than personal identifiers) to protect participant privacy and were independently verified by a second sonographer (Y.Z.) to validate measurement accuracy. Participants were assessed under two conditions (at rest and during PFM contraction) and in seven positions (Figures 1–7) to evaluate the retrovesical angle (RVA), urethral tilt angle (UTA), hiatal area, bladder neck position (BND), cervical position (CP), and rectal ampulla position (RAP). The seven positions included the lithotomy position; drawing-in (participants were asked to pull their abdomen in toward the spine as much as possible, and the contraction was supervised through ultrasonic imaging); head-lift1, which was defined as the position where the head was lifted from the bed just before the shoulder blades were about to leave the surface; head-lift2, which was characterized by the complete lifting of the shoulder blades off the bed; one-leg lift, which was the lithotomy position with one leg hanging over the bed (randomly chosen one leg); hips and knees flexion (relax) HKF(R), which was 90 °flexion of the hips and knees with the legs parallel to each other (supported by tape to ensure participant relaxation); and hips and knees flexion (contraction) HKF(C), which was 90 °flexion of the hips and knees with legs parallel to each other (without supporting), and participants were asked to keep their waist as close to the bed as possible. The drawing-in maneuver was guided with the verbal cue “Gently pull your navel toward your spine,” and a transducer was placed on the lateral abdominal wall to confirm proper activation of the transversus abdominis. There was a 30-s rest between the seven measurements and the positions were performed randomly during testing to prevent fatigue and practice effects.

Participants were asked to empty their bladders before the scan. Transperineal ultrasound with a Mindray Reson8s 4D ultrasound system (Mindray Reson8s (11), Shenzhen, Guangdong, China) was used to evaluate the pelvic floor morphometry of the participants. A transducer (D8-2U Resona 8, Shenzhen, Guangdong, China) was placed on the perineum in a mid-sagittal plane, with a sweep angle of 85° obtained at both the rest position and PFM contraction. The participants were instructed to perform the PFM contractions until they mastered the test correctly and at least two or three contractions were performed with the most effective one being used for evaluation. The hiatal area was measured in the plane of the inferior pubic symphysis as the area enclosed by the pubic bone and the medial borders of the levator ani muscles. The UTA was measured as the angle between the central axis of the urethra and a vertical reference line. The RVA was measured as the angle formed between the proximal urethra and the posterior bladder wall. The positions of the bladder neck, cervix, and rectal ampulla were quantified by their perpendicular distances to the reference line (the inferior margin of the symphysis pubis). Data were acquired during rest and at a maximal voluntary PFM contraction, the latter being defined by the observation of a significant narrowing of the levator hiatus and a distinct cranial lift of the levator plate on ultrasound.

### Sample size calculation

This study included 20 subjects in a preliminary experiment. Head-lift is the most likely position to cause pelvic organ descent according to biomechanics and previous studies (21, 22). In addition, BND is closely correlated with urinary incontinence and has the greatest impact on people’s lives (23–25). Based on these considerations, this study chose the BND parameters in two head-lift positions as the reference for this sample size calculation. The sample size for this study was calculated using PASS software (version 21.0.3). Based on a two-tailed paired *t*-test with an alpha of 0.05 and a power of 0.9, and using the mean ± SD of inter-group differences (0.089 ± 0.240 and 0.114 ± 0.328), the corresponding effect sizes were calculated as 0.371 and 0.346, respectively. These effect sizes yielded required sample sizes of 79 and 90 participants. To ensure sufficient power for the primary analysis, the larger estimate of 90 participants was adopted.

### Statistical analysis

All statistical analyses were performed using the SPSS software (version 26.0) and the R (version 4.4.3). Counting data and measurement data were expressed as (*n*%) and ( $\overset{-}{x}\pm s$ ) respectively. All the data were shown to conform to a normal distribution (Kolmogorov–Smirnova), so that results were compared using paired *t*-tests for within-subject comparisons across different body positions and PFM contraction conditions. To control the risk of type I errors arising from multiple hypothesis testing, the raw *p* values were adjusted for the false discovery rate (FDR) (26). Statistical significance was defined as an FDR-adjusted *p* value < 0.05.

## Results

**Figure 8:**
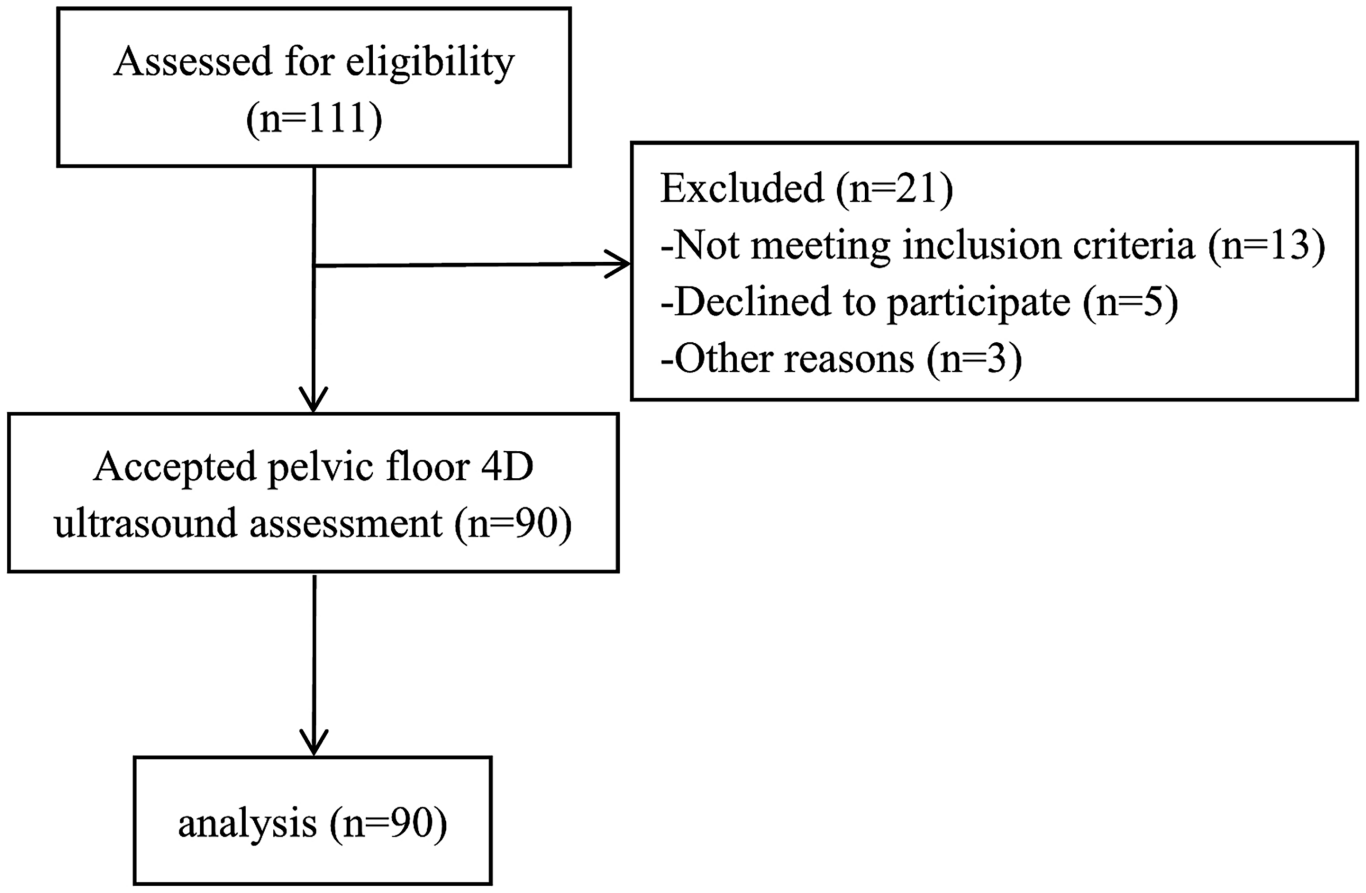
Flow chart.

A total of 111 participants were invited to the study and 90 subjects accepted and completed the ultrasound assessment (Figure 8). Based on the characteristics of the study participants (*n* = 90), a summary of the demographic and clinical data is presented in Table 1. The mean age of the participants was 31.84 ± 3.93 years, with a median of 32 years (Q1–Q3: 29, 35). The mean height, weight, and BMI were 160.77 ± 5.36 cm, 61.01 ± 7.81 kg, and 23.58 ± 2.61 kg/m2, with a median of 160 cm (Q1–Q3: 157, 165), 60.55 kg (Q1–Q3: 55, 66), and 23.83 kg/m2 (Q1–Q3: 21.18, 25.58), respectively. Regarding obstetric characteristics, the mean neonatal weight was 3.30 ± 0.36 kg, with a median of 3.21 kg (Q1–Q3: 3.01, 3.55). The mean gestational weight gain was 11.54 ± 4.34 kg, with a median of 11 kg (Q1–Q3: 8.88, 14.13). The mean gestation was 1.62 ± 0.71, with a median of 1.5 (Q1–Q3: 1, 2), and the mean parity was 1.60 ± 0.67, with a median of 1.5 (Q1–Q3: 1, 2). In terms of education level, 12 participants (13.33%) had 12 years or less of education, while 78 participants (86.67%) had more than 12 years.

**Table 1:** General characteristics of research participants.

Variables	Group	Number (%)	Mean ± SD (0.25–0.5–0.75 quantile)
Age, y			31.84 ± 3.93 (29, 32, 35)
Height, cm			160.77 ± 5.36 (157, 160, 165)
Weight, kg			61.01 ± 7.81 (55, 60.55, 66)
BMI, kg/m^2^			23.58 ± 2.61 (21.18, 23.83, 25.58)
Neonatal weight, kg			3.30 ± 0.36 (3.01, 3.21, 3.55)
Gestational weight gain, kg			11.54 ± 4.34 (8.88, 11, 14.13)
Education	≤12	12 (13.33)	
	>12	78 (86.67)	
Gestation			1.62 ± 0.71 (1, 1.5, 2)
Parity			1.60 ± 0.67 (1, 1.5, 2)

**Figure 9:**
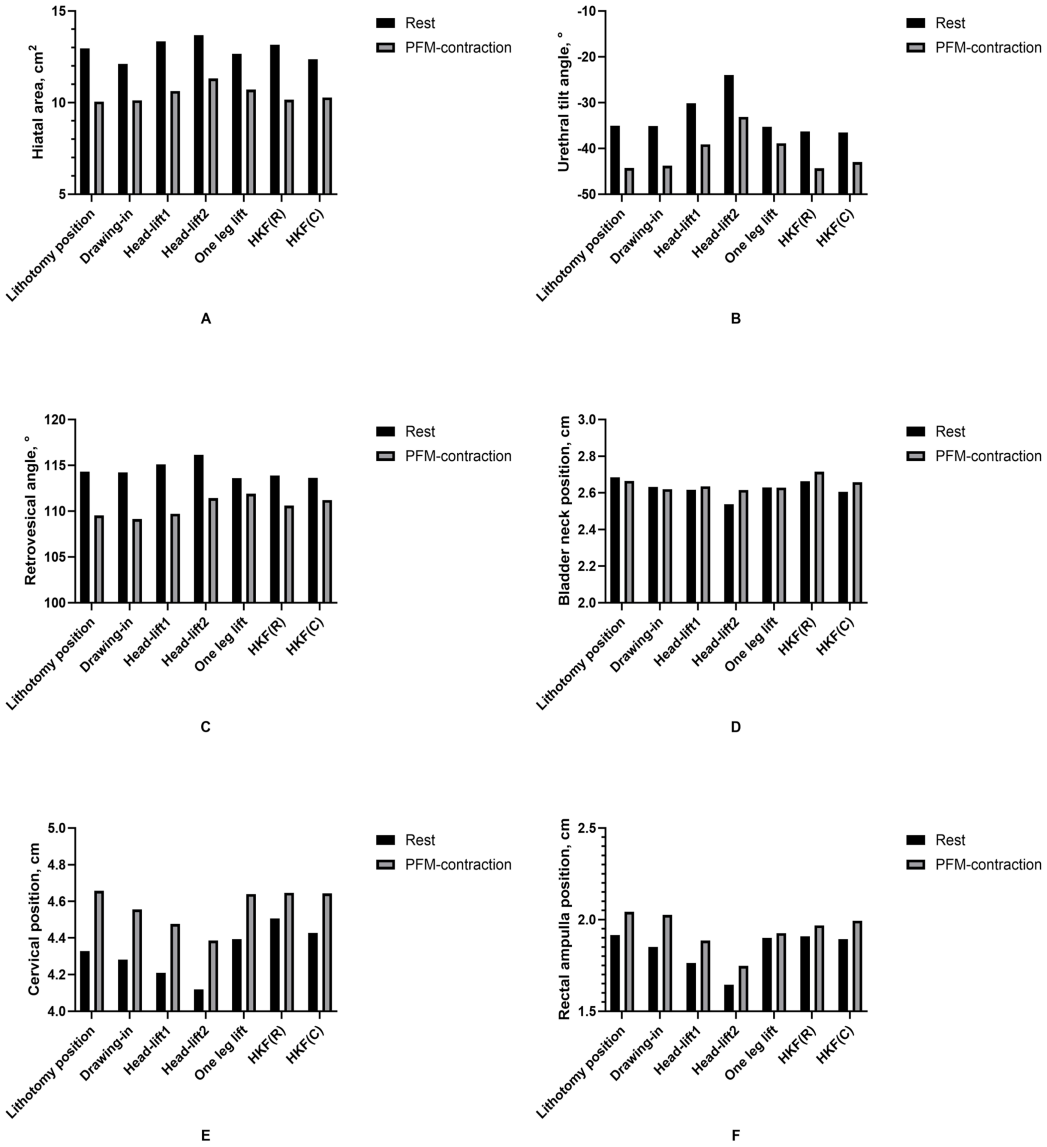
Ultrasonic parameters in two conditions (rest and pelvic floor muscle contraction) and seven positions.

Paired *t*-tests showed that the hiatal area of drawing-in position was lower than that in the lithotomy position, but the hiatal area of head-lift1 and head-lift2 was higher than that in lithotomy position (*Padj <* 0.001, Cohen’s *d*: 0.536, 95%Cl: 0.516, 1.177; *Padj =* 0.027, Cohen’s *d*: −0.253, 95%Cl: −0.711, −0.068; and *Padj <* 0.001, Cohen’s *d*: −0.404, 95%Cl: −1.106, −0.351, respectively). The hiatal area of HKF(C) was lower than that of HKF(R) position (*Padj <* 0.001, Cohen’s *d*: 0.442, 95%Cl: 0.410, 1.148). The UTA of head-lift1 and head-lift2 was all lower than that of lithotomy position (*Padj <* 0.001, Cohen’s *d*: −0.479, 95%Cl: −7.028, −2.750 and *Padj <* 0.001, Cohen’s *d*: −0.801, 95%Cl: −13.959, −8.175, respectively). Except for one leg lift and HKF(C) in RVA, these results showed a significant decreasing trend in hiatal area and RVA and an increasing trend in UTA in PFM contraction (see Tables 2–4, Figures 9A–C).

**Table 2:** The differences between rest and PFM-contraction conditions of hiatal area in seven positions.

Variables	Rest	PFM-contraction	*t*	*p*	*Padj*	Cohen’s *d*	95%Cl	*t*	*p*	*Padj*	Cohen’s *d*	95%Cl
Lithotomy position	12.95 ± 2.45	10.06 ± 1.59	–	–	–	–	–	14.471^c^	<0.001	<0.001	1.525	2.499, 3.294
Drawing-in	12.11 ± 2.30	10.12 ± 1.61	5.088^a^	<0.001	<0.001	0.536	0.516, 1.177	10.806^c^	<0.001	<0.001	1.139	1.620, 2.349
Head-lift1	13.34 ± 2.57	10.63 ± 1.71	−2.404^a^	0.018	0.027	−0.253	−0.711, −0.068	13.469^c^	<0.001	<0.001	1.420	2.311, 3.111
Head-lift2	13.68 ± 2.72	11.33 ± 2.12	−3.831^a^	<0.001	<0.001	−0.404	−1.106, −0.351	11.626^c^	<0.001	<0.001	1.225	1.954, 2.760
One leg lift	12.65 ± 2.44	10.72 ± 1.81	1.643^a^	0.104	0.136	0.173	−0.063, 0.666	10.460^c^	<0.001	<0.001	1.103	1.569, 2.305
HKF(R)	13.15 ± 2.80	10.16 ± 1.79	–	–	–	–	–	14.696^c^	<0.001	<0.001	1.549	2.591, 3.401
HKF(C)	12.37 ± 2.38	10.28 ± 1.72	4.194^b^	<0.001	<0.001	0.442	0.410, 1.148	11.839^c^	<0.001	<0.001	1.248	1.746, 2.450

P primary *p* value; Padj adjusted *p* value; Head-lift1 the shoulder blades are about to leave the bed; Head-lift2 the shoulder blades completely leave off the bed; HKF(R) 90° hips and knees flexion (rest); HKF(C) 90° hips and knees flexion (contraction); ^a^compared with the parameter of lithotomy position in rest condition; ^b^compared with the parameter of HKF(R) in rest condition; ^c^comparison between the rest and PFM-contraction condition.

**Table 3:** The differences between rest and PFM-contraction conditions of urethral tilt angle in seven positions.

Variables	Rest	PFM-contraction	*t*	*p*	*Padj*	Cohen’s *d*	95%Cl	*t*	*p*	*Padj*	Cohen’s *d*	95%Cl
Lithotomy position	−35.06 ± 13.01	−44.28 ± 9.31	–	–	–	–	–	9.757^c^	<0.001	<0.001	1.028	7.344, 11.1
Drawing-in	−35.11 ± 11.58	−43.79 ± 9.51	0.077^a^	0.939	0.952	0.008	−1.375, 1.487	9.941^c^	<0.001	<0.001	1.048	6.943, 10.412
Head-lift1	−30.17 ± 13.71	−39.13 ± 11.12	−4.541^a^	<0.001	<0.001	−0.479	−7.028, −2.750	8.100^c^	<0.001	<0.001	0.854	6.767, 11.166
Head-lift2	−23.99 ± 15.72	−33.11 ± 11.83	−7.603^a^	<0.001	<0.001	−0.801	−13.959, −8.175	6.674^c^	<0.001	<0.001	0.703	6.406, 11.838
One leg lift	−35.31 ± 12.95	−38.89 ± 11.60	0.210^a^	0.834	0.858	0.022	−2.161, 2.672	3.433^c^	<0.001	0.002	0.362	1.507, 5.649
HKF(R)	−36.30 ± 15.00	−44.36 ± 11.50	–	–	–	–	–	7.739^c^	<0.001	<0.001	0.816	5.987, 10.124
HKF(C)	−36.51 ± 14.28	−42.97 ± 11.58	0.216^b^	0.829	0.858	0.023	−1.730, 2.153	6.203^c^	<0.001	<0.001	0.654	4.388, 8.523

P primary *p* value; Padj adjusted *p* value; Head-lift1 the shoulder blades are about to leave the bed; Head-lift2 the shoulder blades completely leave off the bed; HKF(R) 90° hips and knees flexion (rest); HKF(C) 90° hips and knees flexion (contraction); ^a^compared with the parameter of lithotomy position in rest condition; ^b^compared with the parameter of HKF(R) in rest condition; ^c^comparison between the rest and PFM-contraction condition.

**Table 4:** The differences between rest and PFM-contraction conditions of retrovesical angle in seven positions.

Variables	Rest	PFM-contraction	*t*	*p*	*Padj*	Cohen’s *d*	95%Cl	*t*	*p*	*Padj*	Cohen’s *d*	95%Cl
Lithotomy position	114.29 ± 10.03	109.52 ± 10.79	–	–	–	–	–	3.584^c^	0.001	0.001	0.378	2.124, 7.409
Drawing-in	114.22 ± 9.30	109.12 ± 10.95	0.058^a^	0.954	0.954	0.006	−2.229, 2.362	4.406^c^	<0.001	<0.001	0.464	2.800, 7.4
Head-lift1	115.11 ± 8.54	109.71 ± 10.22	−0.670^a^	0.505	0.577	−0.071	−3.261, 1.617	6.540^c^	<0.001	<0.001	0.689	3.759, 7.041
Head-lift2	116.13 ± 10.46	111.42 ± 10.12	−1.361^a^	0.177	0.224	−0.143	−4.538, 0.849	3.725^c^	<0.001	<0.001	0.393	2.198, 7.224
One leg lift	113.60 ± 10.74	111.89 ± 9.20	0.494^a^	0.623	0.690	0.052	−2.085, 3.462	1.294^c^	0.199	0.247	0.136	−0.917, 4.339
HKF(R)	113.89 ± 11.48	110.59 ± 10.75	–	–	–	–	–	2.664^c^	0.009	0.015	0.281	0.838, 5.762
HKF(C)	113.62 ± 10.33	111.20 ± 10.79	0.214^b^	0.831	0.858	0.023	−2.206, 2.739	1.838^c^	0.069	0.094	0.194	−0.197, 5.041

P primary *p* value; Padj adjusted *p* value; Head-lift1 the shoulder blades are about to leave the bed; Head-lift2 the shoulder blades completely leave off the bed; HKF(R) 90° hips and knees flexion (rest); HKF(C) 90° hips and knees flexion (contraction); ^a^compared with the parameter of lithotomy position in rest condition; ^b^compared with the parameter of HKF(R) in rest condition; ^c^comparison between the rest and PFM-contraction condition.

The bladder neck position descended in drawing-in, head-lift1, head-lift2, and one leg lift (*Padj =* 0.005, Cohen’s *d*: 0.324, 95%Cl: 0.019, 0.087; *Padj =* 0.002, Cohen’s *d*: 0.357, 95%Cl: 0.028, 0.107; *Padj <* 0.001, Cohen’s *d* = 0.496, 95%Cl: 0.085, 0.209, and *Padj =* 0.018, Cohen’s *d*: 0.273, 95%Cl: 0.013, 0.098, respectively), the differences remained significant during PFM contraction (*Padj <* 0.001, Cohen’s *d*: 0.398, 95%Cl: 0.031, 0.099; *Padj =* 0.014, Cohen’s *d*: 0.285, 95%Cl: 0.013, 0.085; *Padj =* 0.023, Cohen’s *d*: 0.262, 95%Cl: 0.014, 0.125; and *Padj =* 0.033, Cohen’s *d*: 0.244, 95%Cl: 0.008, 0.106, respectively). The bladder neck position descended in HKF(C) position and returned to approximately the baseline position during PFM contraction in HKF(R) (*Padj <* 0.001, Cohen’s *d*: 0.403, 95%Cl = 0.028, 0.089; and *Padj =* 0.822, Cohen’s *d*: 0.033, 95%Cl: −0.024, 0.033, respectively) (Table 5). The cervical position descended in head-lift1, head-lift1, (*Padj =* 0.032, Cohen’s *d*: 0.245, 95%Cl: 0.017, 0.219; *Padj <* 0.001, Cohen’s *d*: 0.438, 95%Cl: 0.109, 0.309, respectively), and rose during PFM contraction in lithotomy, drawing-in, head-lift1, and one leg lift (*Padj <* 0.001, Cohen’s *d*: −1.116, 95%Cl: −0.392, −0.268; *Padj <* 0.001, Cohen’s *d*: −0.519, 95%Cl: −0.320, −0.136; *Padj =* 0.011, Cohen’s *d*: −0.297, 95%Cl: −0.254, −0.044; and *Padj <* 0.001, Cohen’s *d*: −0.477, 95%Cl: −0.449, −0.175, respectively). The cervical position descended in HKF(C) position (not significant) and rose to a higher position compared with that in HKF(R) position (*Padj =* 0.006, Cohen’s *d*: −0.320, 95%Cl: −0.226, −0.047) (Table 6). The RAP descended in drawing-in, head-lift1, and head-lift2 (*Padj =* 0.018, Cohen’s *d*: 0.273, 95%Cl: 0.015, 0.114; *Padj <* 0.001, Cohen’s *d*: 0.562, 95%Cl: 0.096, 0.209; and *Padj <* 0.001, Cohen’s *d*: 0.894, 95%Cl: 0.208, 0.335, respectively), and the differences remained significant during PFM contraction in head-lift2 position (*Padj <* 0.001, Cohen’s *d*: 0.521, 95%Cl: 0.101, 0.237). The RAP descended in HKF(C) position (not significant) and rose to a higher position compared with that in HKF(R) position (*Padj =* 0.005, Cohen’s *d*: −0.325, 95%Cl: −0.140, −0.030) (Table 7, Figures 9D–F).

**Table 5:** The differences between rest and PFM-contraction conditions of bladder neck position in seven positions.

Variables	Rest	PFM-contraction	*t*	*p*	*Padj*	Cohen’s *d*	95%Cl	*t*	*p*	*Padj*	Cohen’s *d*	95%Cl
Lithotomy position	2.68 ± 0.26	2.66 ± 0.26	–	–	–	–	–	1.271^c^	0.207	0.253	0.134	−0.011, 0.052
Drawing-in	2.63 ± 0.28	2.62 ± 0.26	3.072^a^	0.003	0.005	0.324	0.019, 0.087	3.772^c^	<0.001	<0.001	0.398	0.031, 0.099
Head-lift1	2.62 ± 0.28	2.64 ± 0.27	3.385^a^	0.001	0.002	0.357	0.028, 0.107	2.708^c^	0.008	0.014	0.285	0.013, 0.085
Head-lift2	2.54 ± 0.36	2.62 ± 0.34	4.703^a^	<0.001	<0.001	0.496	0.085, 0.209	2.487^c^	0.015	0.023	0.262	0.014, 0.125
One leg lift	2.63 ± 0.29	2.63 ± 0.29	2.593^a^	0.011	0.018	0.273	0.013, 0.098	2.312^c^	0.023	0.033	0.244	0.008, 0.106
HKF(R)	2.66 ± 0.28	2.72 ± 0.29	–	–	–	–	–	−5.32^d^	<0.001	<0.001	−0.561	−0.073, −0.033
HKF(C)	2.60 ± 0.27	2.66 ± 0.26	3.827^b^	<0.001	<0.001	0.403	0.028, 0.089	0.315^d^	0.753	0.822	0.033	−0.024, 0.033

P primary *p* value; Padj adjusted *p* value; Head-lift1 the shoulder blades are about to leave the bed; Head-lift2 the shoulder blades completely leave off the bed; HKF(R) 90° hips and knees flexion (rest); HKF(C) 90° hips and knees flexion (contraction); ^a^compared with the parameter of lithotomy position in rest condition; ^b^compared with the parameter of HKF(R) in rest condition; ^c^compared with the parameter of HKF(R) in rest condition; ^d^compared with the parameter of HKF(R) in rest condition.

**Table 6:** The differences between rest and PFM-contraction conditions of cervical position in seven positions.

Variables	Rest	PFM-contraction	*t*	*p*	*Padj*	Cohen’s *d*	95%Cl	*t*	*p*	*Padj*	Cohen’s *d*	95%Cl
Lithotomy position	4.33 ± 0.94	4.66 ± 0.89	–	–	–	–	–	−10.588^c^	<0.001	<0.001	−1.116	−0.392, −0.268
Drawing-in	4.28 ± 0.94	4.56 ± 0.88	1.772^a^	0.080	0.106	0.187	−0.006, 0.099	−4.921^c^	<0.001	<0.001	−0.519	−0.320, −0.136
Head-lift1	4.21 ± 1.00	4.48 ± 0.91	2.325^a^	0.022	0.032	0.245	0.017, 0.219	−2.822^c^	0.006	0.011	−0.297	−0.254, −0.044
Head-lift2	4.12 ± 0.99	4.39 ± 0.93	4.158^a^	<0.001	<0.001	0.438	0.109, 0.309	−1.053^c^	0.295	0.354	−0.111	−0.169, 0.052
One leg lift	4.39 ± 0.99	4.64 ± 0.98	−1.431^a^	0.156	0.201	−0.151	−0.157, 0.025	−4.521^c^	<0.001	<0.001	−0.477	−0.449, −0.175
HKF(R)	4.51 ± 1.14	4.65 ± 0.98	–	–	–	–	–	−2.773^d^	0.007	0.012	−0.292	−0.241, −0.040
HKF(C)	4.43 ± 1.01	4.64 ± 0.98	2.087^b^	0.040	0.055	0.220	0.004, 0.154	−3.039^d^	0.003	0.006	−0.320	−0.226, −0.047

P primary *p* value; Padj adjusted *p* value; Head-lift1 the shoulder blades are about to leave the bed; Head-lift2 the shoulder blades completely leave off the bed; HKF(R) 90° hips and knees flexion (rest); HKF(C) 90° hips and knees flexion (contraction); ^a^compared with the parameter of lithotomy position in rest condition; ^b^compared with the parameter of HKF(R) in rest condition; ^c^compared with the parameter of HKF(R) in rest condition; ^d^compared with the parameter of HKF(R) in rest condition.

**Table 7:** The differences between rest and PFM-contraction conditions of rectal ampulla position in seven positions.

Variables	Rest	PFM-contraction	*t*	*p*	*Padj*	Cohen’s *d*	95%Cl	*t*	*p*	*Padj*	Cohen’s *d*	95%Cl
Lithotomy position	1.92 ± 0.29	2.04 ± 0.30	–	–	–	–	–	−6.045^c^	<0.001	<0.001	−0.637	−0.169, −0.085
Drawing-in	1.85 ± 0.28	2.03 ± 0.42	2.592^a^	0.011	0.018	0.273	0.015, 0.114	−2.500^c^	0.014	0.022	−0.264	−0.197, −0.023
Head-lift1	1.76 ± 0.28	1.89 ± 0.28	5.334^a^	<0.001	<0.001	0.562	0.096, 0.209	1.011^c^	0.315	0.372	0.107	−0.029, 0.089
Head-lift2	1.64 ± 0.30	1.75 ± 0.33	8.483^a^	<0.001	<0.001	0.894	0.208, 0.335	4.940^c^	<0.001	<0.001	0.521	0.101, 0.237
One leg lift	1.90 ± 0.30	1.93 ± 0.31	0.561^a^	0.576	0.648	0.059	−0.042, 0.076	−0.285^c^	0.776	0.834	−0.030	−0.084, 0.063
HKF(R)	1.91 ± 0.31	1.97 ± 0.28	–	–	–	–	–	−2.376^d^	0.020	0.029	−0.250	−0.109, −0.010
HKF(C)	1.89 ± 0.31	1.99 ± 0.33	0.799^b^	0.426	0.495	0.084	−0.023, 0.054	−3.081^d^	0.003	0.005	−0.325	−0.140, −0.030

P primary *p* value; Padj adjusted *p* value; Head-lift1 the shoulder blades are about to leave the bed; Head-lift2 the shoulder blades completely leave off the bed; HKF(R) 90° hips and knees flexion (rest); HKF(C) 90° hips and knees flexion (contraction); ^a^compared with the parameter of lithotomy position in rest condition; ^b^compared with the parameter of HKF(R) in rest condition; ^c^compared with the parameter of HKF(R) in rest condition; ^d^compared with the parameter of HKF(R) in rest condition.

## Discussion

Abdominal exercises are recommended for treating diastasis recti abdominis (DRA) and low back pain (14, 15). In postpartum rehabilitation, exercises focusing on the transversus abdominis are more commonly described in the literature than those targeting the rectus abdominis (27). Due to their higher intensity, movements such as sit-ups and leg raise sit-ups are generally not recommended in early postpartum exercise programs (28). Therefore, drawing-in and lower-intensity activities like head-lifts and leg-lifts have been adopted in postpartum training protocols and were selected as part of the research targets of this study (29, 30). Additionally, two positions were included as controls, namely the lithotomy position and HKF(R). This is because pelvic position may influence pelvic floor parameters, as suggested in earlier work (31), and because the pelvic orientation and reference line (inferior margin of the pubic symphysis) change when the hips and knees are flexed to 90°.

First, the participants performed PFM contractions effectively in all seven positions, as indicated by reductions in hiatal area, RVA, and increase in UTA (Tables 2–4, Figures 9A–C). Further, Figures 9D–F also shows that nearly all pelvic organs were positioned higher during PFM contraction compared to the resting state.

The decrease in hiatal area during abdominal drawing-in suggests that this maneuver readily activates the pelvic floor muscles. This aligns with earlier studies, even though we specifically recruited participants able to perform PFM contractions without abdominal muscle interference (32–35). The co-contraction of the pelvic floor and abdominal muscles might be related to their neural connections. Research has shown that in cats and rhesus monkeys, the pelvic floor and rectus abdominis muscles share common projections in the brainstem (36, 37). Stimulation of the human S3 nerve root also activates both the abdominal and pelvic floor motor cortex (38). Djivoh et al. demonstrated that transversus abdominis contraction increases intra-abdominal pressure (16), and this study also similarly observed a descent of the bladder neck and rectal ampulla during the drawing-in maneuver. When PFM contraction was added, the cervix and rectal ampulla elevated, although the bladder neck remained lower than at rest, indicating that PFM contraction can provide protection during abdominal drawing-in.

Both head-lift (head-lift1 and head-lift2) maneuvers exerted considerable pressure on the pelvic floor, not only enlarging the hiatal area but also displacing the bladder neck, cervix, and rectal ampulla downward, particularly during head-lift2. Similar to the findings of this study, previous studies have reported increased pressure during head-lift exercises (21, 22, 39). When PFM contraction was performed concurrently, all three organs ascended, although the rectal ampulla remained below the resting level during head-lift2. Compared to head-lift1, PFM contraction failed to fully counteract the pressure exerted on pelvic floor organs by the increased intra-abdominal pressure resulting from head-lift2.

One-leg lift showed a minor effect on the position of the bladder neck, cervix, or rectal ampulla. Compared to other lower-limb exercises, one-leg lift appeared to generate less abdominal muscle activity and therefore less pressure on the pelvic floor (40). Additionally, PFM contraction did not notably alter organ position in this posture, possibly because contracting the pelvic floor is more challenging in this position.

HKF(C) also induced descent of all three organs, with statistically significant changes at the bladder neck and cervix. Unlike the active rectus abdominis contraction in head-lift2, HKF(C) involves isometric flexion maintained by posterior pelvic tilt against increasing resistance (41). This position also significantly increased intra-abdominal pressure, likely due to co-contraction of the transversus abdominis and internal/external obliques to stabilize the pelvis (41). When PFM contraction was added, all three organs returned nearly to their starting positions. This restoration may be linked to hip adductor activation in HKF(C), as such activation could promote greater PFM engagement (42).

There were also some limitations in this study. First, the single-center design and only included women who delivered vaginally at our institution. Furthermore, the exclusion of women unable to perform isolated PFM contractions may collectively limit the generalizability of these findings to diverse populations or healthcare settings. Furthermore, the study did not take into account factors such as parity, the number of fetuses, postpartum interval, and instrumental deliveries, which may contribute to more severe pelvic floor damage, thereby impairing the ability to counteract increased intra-abdominal pressure and subsequently compromising the accuracy of these results. Additionally, technical limitations restrict the observation to the resting pelvic floor, making the dynamic changes during movement unclear. Finally, this study lacked long-term follow-up data, but a new trial was conducted to observe long-term effects of abdominal exercise on both DRA and pelvic floor muscles.

## Conclusion

Drawing-in, head-lift, and flexion of the hips and knees were associated with immediate pelvic organ descent and hiatal area enlargement in this study, with the head-lift2 maneuver showing a relatively higher degree of mechanical loading. The simultaneous contraction of the pelvic floor muscles during these maneuvers correlated with an immediate reduction in pelvic organ descent. Within the context of these observed immediate effects, the integration of transversus abdominis activation can be considered for its potential to co-activate the PFM. For individuals regularly performing high-intensity maneuvers such as head-lift2, periodic pelvic floor ultrasound could be a useful tool to monitor structural changes.

## Data Availability

The original contributions presented in the study are included in the article/supplementary material, further inquiries can be directed to the corresponding author.
